# Dysfunction of cross-frequency phase-phase coupling in primary dysmenorrhea: a resting magnetoencephalographic study

**DOI:** 10.1186/1471-2202-13-S1-P168

**Published:** 2012-07-16

**Authors:** Pin-Shiuan Lee, Yong-Sheng Chen, Jen-Chuen Hsieh, Li-Fen Chen

**Affiliations:** 1 Institute of Biomedical Informatics, National Yang-Ming University, Taipei 112, Taiwan; 2 Department of Computer Science, National Chiao Tung University, Hsinchu 300, Taiwan; 3 Institute of Brain Science, National Yang-Ming University, Taipei 112, Taiwan; 4 Integrated Brain Research Laboratory, Department of Medical Research and Education, Taipei Veterans General Hospital, Taipei 112, Taiwan

## 

Cross-frequency synchronization between neuronal ensembles has been studied recently, which is related to coupling between neuronal oscillations of different frequency contents [1]. This study aimed at the investigation of how cross-frequency phase-phase coupling of local network during rest is modulated by pain experience. Ten primary dysmenorrhea (PDM) females suffering lower abdominal *pain during* menstrual phase *and ten age-matched healthy females were enrolled.* T*hree-minute eye-open resting* magnetoencephalographic (MEG) signals of *each individual during menstrual phase were recorded* using a 306-channel MEG system. For each channel, synchronization value of cross-frequency coupling was estimated by calculating phase-locking statistics of phase differences between two frequency bands, including 2, 4, 8, 12, 16, 24, 32, and 40 Hz, respectively. The results of one-sample binomial test showed that in low alpha/beta (8 / 16 Hz) oscillations, the PDM group displayed coupling in the medial parietal area whereas no coupling in the control group (Figure 1(a)). On the other hand, coupling at the prefrontal area found in the NC group was not found in the PDM group. These two regions have been reported as part of resting state networks [2]. No difference of coupling between the PDM and control groups was found in other combinations of frequency pairs, for instance, high alpha/beta (Figure 1(b)) and beta/gamma (Figure 1(c)).

**Figure 1:**
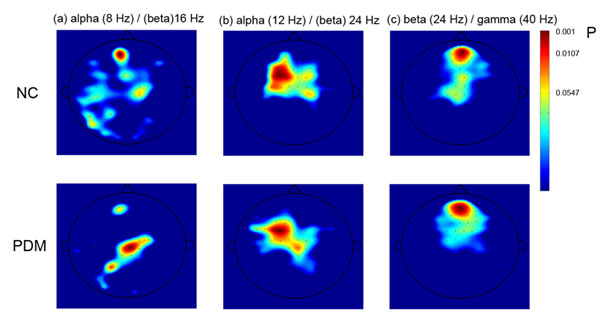
Topography of different pairs of cross-frequency coupling between PDM and normal controls. The color represents significant level of phase-locking statistics at the respective channel for each group.

## Conclusion

Our findings implicate that pain experience may modulate phase-phase coupling of alpha/beta oscillation, which might disrupt integration between nearby neural population in the human neocortex at rest.
